# Case report: Surgical treatment and literature review of a recurrent case of glucagonoma

**DOI:** 10.3389/fonc.2024.1437102

**Published:** 2024-08-26

**Authors:** Zhipeng Liu, Faji Yang, Yijie Hao, Qirong Jiang, Yisu Zhang, Qixuan Zheng, Yupeng Jiang, Jun Lu, Hengjun Gao

**Affiliations:** ^1^ Department of Hepatobiliary Surgery, Shandong Provincial Hospital, Shandong First Medical University, Jinan, China; ^2^ Department of Hepatobiliary Surgery, Shandong Provincial Hospital, Shandong University, Jinan, China

**Keywords:** glucagonoma, pancreatic neuroendocrine tumor, surgery, liver metastasis 2, rare diseases

## Abstract

A 40-year-old male patient was admitted due to abdominal distension and discomfort in the upper abdomen persisting for three days. Enhanced CT of the upper abdomen revealed an irregularly dense soft tissue area in the body and tail of the pancreas, approximately 7.6 × 3.1 cm in size, with blurred boundaries, and indistinct separation from the splenic artery and vein. Multiple liver lesions of varying sizes and slightly lower densities were also observed. Liver tumor biopsy considering a neuroendocrine tumor G2, combined with the medical history, led to a diagnosis of pancreatic neuroendocrine tumor G2 with liver metastasis. Physical examination showed mild tenderness in the upper abdomen but no other significant positive signs. During treatment, the patient developed multiple red papular rashes around the mouth, on both lower limbs, and the perineum, accompanied by itching. The glucagon level was 1138.3 pg/L. The patient underwent resection of the pancreatic body and tail, splenectomy, partial liver tumor resection, and cholecystectomy. Within five days post-surgery, the skin lesions began to crust and flake off. On the 14th day post-surgery, the serum glucagon level was rechecked at 136.4 pg/L. As of April 2024, progression of liver lesions was noted, with no significant skin symptoms during the period.

## Background

Glucagonoma (GCGN) is a rare pancreatic neuroendocrine tumor (pancreatic neuroendocrine tumor, pNET) originating from pancreatic islet A2 cells, whereby the islet cells secrete abundant glucagon (1). The estimated annual incidence of glucagonoma is ~1 case per 20,000,000 individuals (2). Consequently, there is limited research and reporting on GCGN within China. GCGN typically occurs in the body and tail of the pancreas and is often accompanied by liver metastasis (3). The classic clinical features of GCGN include migratory necrolytic erythema, perioral dermatitis/glossitis, and diabetes. The low incidence rate and the lack of early characteristic symptoms can easily lead to misdiagnosis of GCGN, adversely affecting diagnosis, treatment, and prognosis. This paper aims to report on a case of GCGN with liver metastasis treated by the Department of Hepatobiliary Surgery at the East Hospital of Shandong First Medical University and provides a detailed review of the treatment process.

## Case introduction

The patient, a 40-year-old male, was admitted due to upper abdominal distension and discomfort persisting for three days.

**Figure 1:**
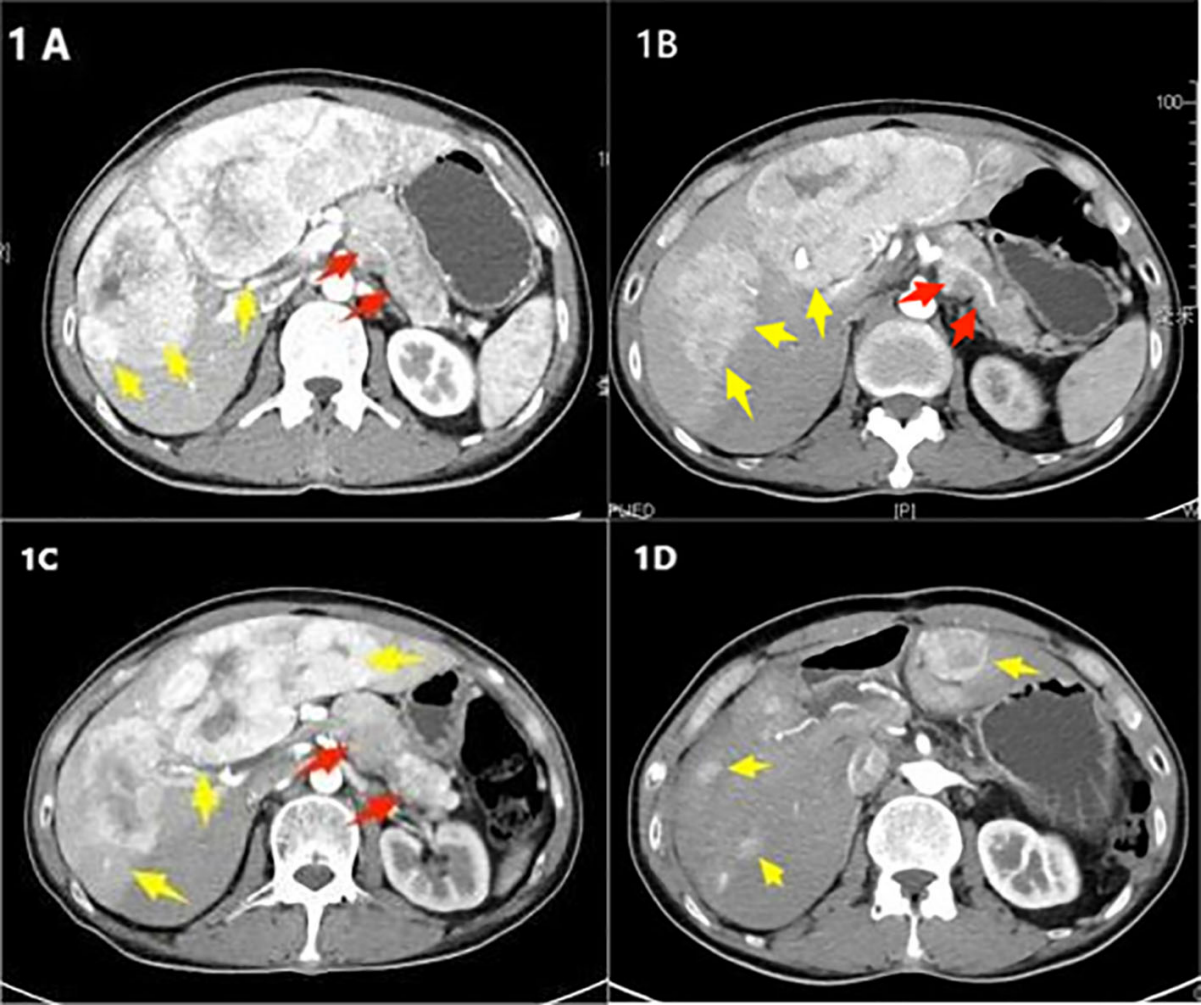
**(A)** Initial treatment in May 2020, an enhanced CT scan of the upper abdomen shows an irregular area of increased soft tissue density in the body and tail of the pancreas, approximately 7.6 × 3.1 cm (indicated by a red arrow). Multiple lesions are present in the liver (indicated by yellow arrows). **(B)** Follow-up in August 2022, an enhanced CT scan of the upper abdomen reveals the irregular area of increased soft tissue density in the body and tail of the pancreas has changed slightly, measuring about 7.3 × 3.6 cm (red arrow). Multiple liver lesions are observed, with the largest located in the left lobe of the liver, approximately 17 × 13 × 6.3 cm. The lesion in the right lobe has increased in size compared to earlier, measuring about 3.9 × 4.0 cm (yellow arrow). **(C)** Preoperative check in February 2023, an enhanced CT scan of the upper abdomen shows the irregular soft tissue density lesion in the body and tail of the pancreas, approximately 7.5 × 4.0 cm (red arrow). The largest lesion in the liver, located in the left lobe, measures approximately 17.5 × 13.5 × 7 cm, showing progression from the previous scans (yellow arrow). **(D)** Clinic follow-up in August 2023, an enhanced CT scan of the upper abdomen after surgery shows the absence of the body and tail of the pancreas, spleen, gallbladder, and parts of the liver. Multiple nodular lesions of slightly lower density are observed in the remaining liver parenchyma, with the largest lesion measuring about 3.1 × 4.2 cm (yellow arrow).

**Figure 2:**
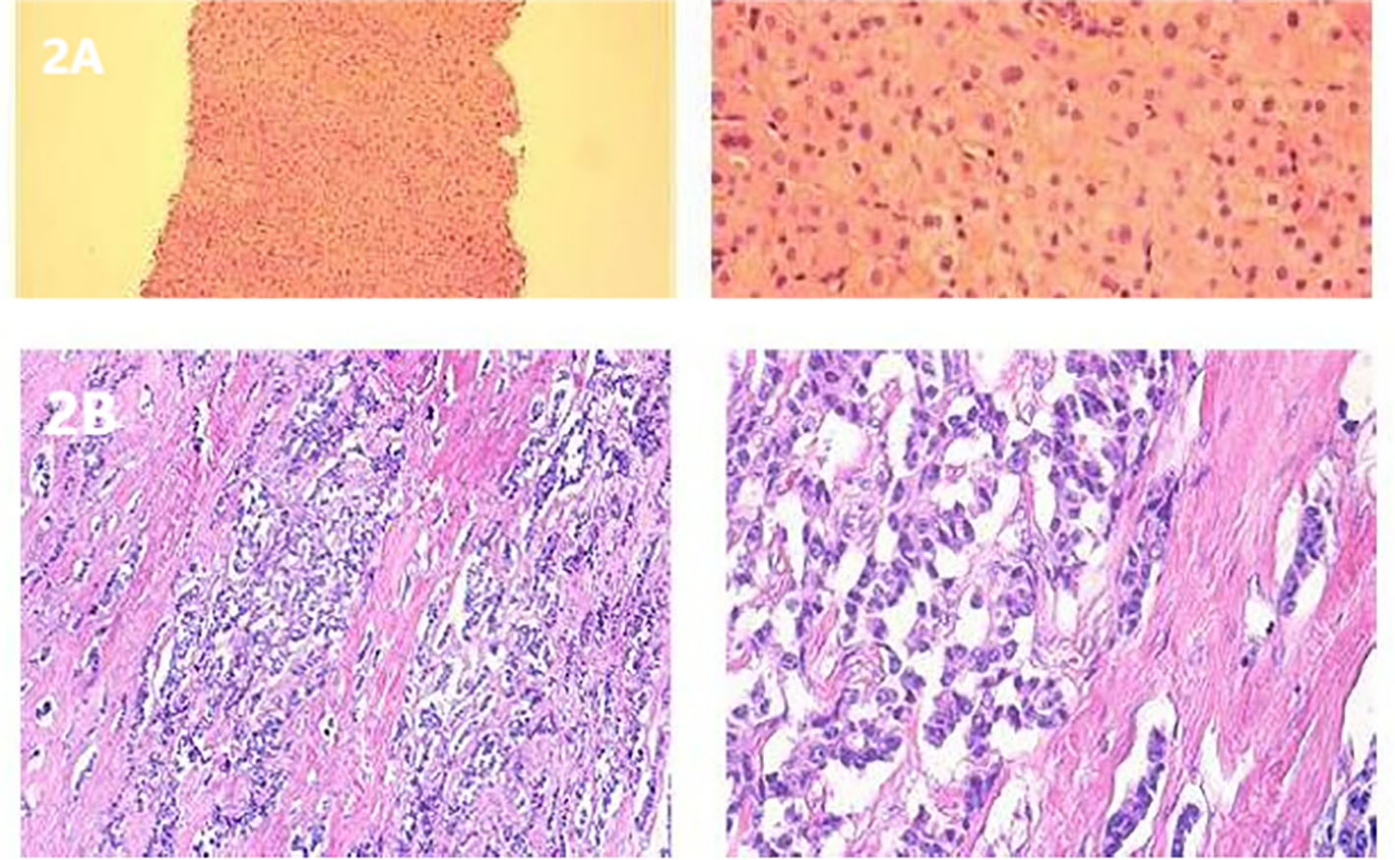
**(A)** H&E staining of a liver metastasis. **(B)** Postoperative histological staining of a pancreatic tumor section.

In his initial treatment in May 2020, an abdominal ultrasound was performed due to abdominal pain, suggesting a pancreatic mass lesion. Subsequently, he was admitted for a detailed enhanced CT scan of the upper abdomen, which revealed an irregularly dense soft tissue area in the body and tail of the pancreas, measuring approximately 7.6 × 3.1 cm, with blurred boundaries, and unclear separation from the splenic artery and vein. Multiple liver lesions of varying sizes and slightly lower densities were also noted (**Figure 1A**) A liver tumor biopsy considering a neuroendocrine tumor G2, immunohistochemistry showed positive for Syn and CgA, weakly positive for CD56, and negative for Hepatocyte, Glypican-3, Arginase-1, CK7, with a Ki-67 of 3% (**Figure 2A**). Based on his medical history, he was diagnosed with pancreatic neuroendocrine tumor G2 with liver metastasis. His treatment included Transarterial Embolization (TAE) under local anesthesia on May 8, 2020, angiography showed a left liver tumor approximately 10 × 7 cm and multiple right liver tumors, the largest being about 6 × 5 cm. He was discharged the day after the procedure without significant discomfort.

On December 24, 2020, a follow-up enhanced CT scan of the upper abdomen showed an irregularly dense soft tissue area in the pancreatic body and tail, approximately 7.8 × 3.1 cm, with still blurred edges invading the splenic artery and vein, compared to May 1, 2020, with enlarged abdominal lymph nodes and multiple liver lesions of varying sizes and slightly lower densities observed. He was regularly injected with Octreotide LAR (Sandostatin) 30 mg intramuscularly. He received three TAE treatments in conjunction with regular Octreotide LAR 30 mg injections every three weeks.

On August 11, 2022, an enhanced CT scan of the upper abdomen showed an irregularly dense area in the pancreatic body and tail, approximately 7.3 × 3.6 cm, with blurred boundaries and indistinct separation from the splenic artery and vein. Multiple liver lesions of varying sizes and uneven densities were observed, with significant ring enhancement in the largest lesion located in the left lobe of the liver, measuring about 17 × 13 × 6.3 cm. The lesion in the upper segment of the right anterior lobe had increased in size, measuring approximately 3.9 × 4.0 cm (**Figures 1B**). Given the progression of liver metastases, the patient received Octreotide LAR injections combined with oral Sunitinib 250 mg daily.

**Figure 3:**
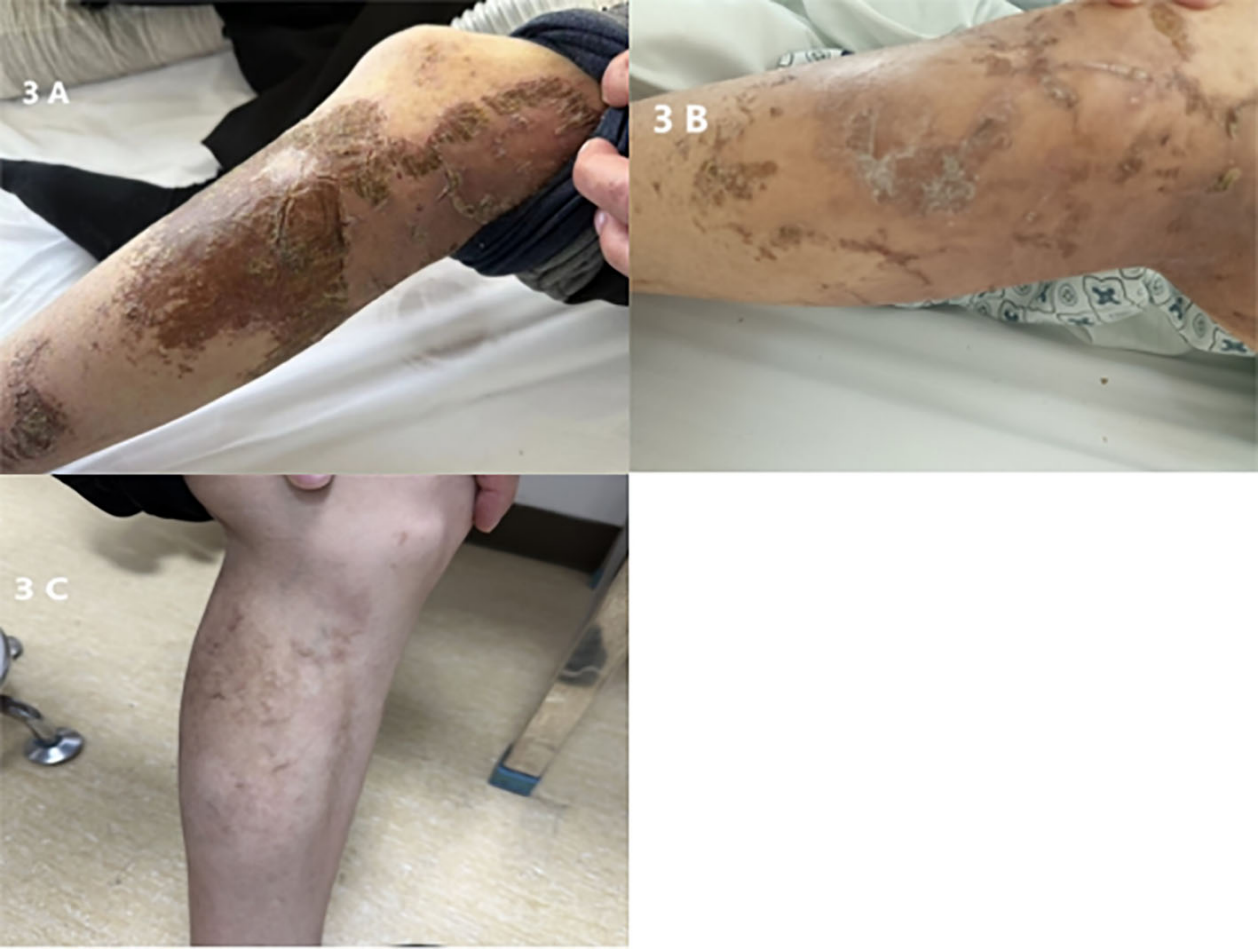
**(A)** Preoperative lower limb skin lesion. **(B)** Lower limb skin lesion, 5 days postoperative. **(C)** Lower limb skin lesion, six months postoperative.

In February 2023, the patient developed multiple red papular rashes without a clear cause, accompanied by itching. There were multiple scattered rashes on the mouth, buttocks, back, and both lower limbs, with those on the legs being larger, approximately 5 cm in diameter, some of which were confluent (**Figure 3A**). Physical examination showed no other significant abnormalities; blood glucose levels were normal. A biopsy of the skin lesions was consistent with necrolytic migratory erythema. A follow-up enhanced CT of the upper abdomen showed an irregularly dense lesion in the pancreatic body and tail, approximately 7.5 × 4.0 cm, with blurred boundaries and indistinct separation from the splenic artery and vein. Multiple liver lesions of varying sizes and densities were observed, the largest in the left lobe of the liver measuring approximately 17.5 × 13.5 × 7 cm, showing progression (**Figure 1C**). Glucagon level was 1138.3 pg/L, neuron-specific enolase was 115.30 ng/ml, and other tumor markers were normal.

**Figure 4:**
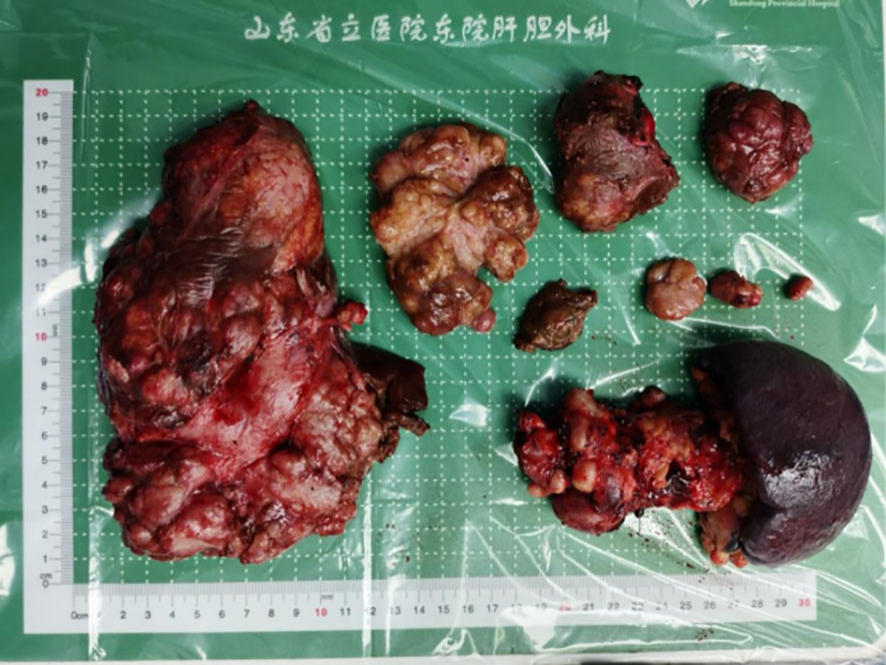
February 23, 2023 - Resection of the pancreatic body and tail, splenectomy, and hepatic tumor resection (approximately 90%), postoperative specimen.

On February 23, 2023, the patient underwent resection of the pancreatic body and tail, splenectomy, partial hepatectomy, and cholecystectomy under general anesthesia (**Figure 4**). Postoperative pathology confirmed a pancreatic neuroendocrine tumor, G2, measuring 5 × 3.2 cm; no tumors were found in the surrounding pancreatic lymph nodes (total 6) or splenic tissue. Liver tumors (numbers 1 to 5) were all metastatic neuroendocrine tumors, G2, measuring respectively 9 × 7 cm, 7.5 × 6 cm, two sites of 2.9 × 2.4 cm and 2 × 1.4 cm, 4 × 2.2 cm, and two sites of 2.5 × 1.2 cm and 1.7 × 1.5 cm. Immunohistochemistry for sample 1 was negative for CK7, positive for Syn, CgA, diffusely positive for SSTR2, and sporadically positive for PHH3, with a Ki-67 hotspot of 10%; sample 21 was negative for P53, sporadically positive for PHH3, and a Ki-67 hotspot of 10% (**Figure 2B**). Five days post-surgery, the patient’s skin lesions began to crust and flake off (**Figure 3B**). On day 14 post-surgery, a serum glucagon level recheck was 136.4 pg/L, close to normal levels. The patient’s skin symptoms largely resolved during the hospital stay, with a total hospitalization time of 24 days.

After discharge, the patient underwent regular follow-ups and continued with Octreotide LAR adjunct therapy. On August 10, 2023, a clinic follow-up enhanced CT of the upper abdomen showed postoperative absence of the pancreatic body and tail, spleen, gallbladder, and part of the liver. Multiple nodular slightly low-density lesions were observed in the remaining liver parenchyma, with the larger lesion in the upper posterior segment of the right liver measuring approximately 3.1 × 4.2 cm. Enlarged lymph nodes in the abdominal and retroperitoneal areas were more pronounced than before, considered metastatic changes (**Figure 1D**). Serum glucagon levels were 166.2 pg/L. The patient continued with Octreotide LAR, Sunitinib, and TAE treatment. As of April 2024, the patient’s general condition was good, with no significant skin symptoms during the period (**Figure 3C**).

## Discussion

Neuroendocrine tumors (NETs) originate from the neuroendocrine system, which is widely distributed throughout the human body and represents a class of heterogeneous tumors. The stomach, intestines, and pancreas are the most common sites for NETs, particularly the small intestine, cecum, appendix, and pancreas (4). Pancreatic neuroendocrine tumors (pNETs) are categorized into functional pancreatic neuroendocrine tumors (FpNETs) and non-functional pancreatic neuroendocrine tumors (uFpNETs) based on whether the tumor is functional. FpNETs account for 34.4% of all pNETs, with 94.8% being insulinomas. Other functional pancreatic neuroendocrine tumors, including vasoactive intestinal peptide tumors (VIPomas), glucagonomas (GCGN), and somatostatinomas, are collectively referred to as rare pancreatic neuroendocrine tumors. GCGN was first reported by Becker in 1942 (5), and its skin manifestation, known as necrolytic migratory erythema (NME), was named by Wilkinson in 1973 (6). In 1974, Mallinson coined the term “glucagonoma syndrome” for a constellation of symptoms including NME, perioral dermatitis, glossitis, diabetes, anemia, and weight loss, attributed to the over-secretion of glucagon (7).

NME is a typical paraneoplastic skin manifestation of glucagonoma, present as the sole clinical symptom in about 70% of patients (8). Typical characteristics of NME lesions include itching, pain, and well-defined erythema (2, 9), most commonly starting in the lower limbs but also affecting the face, upper limbs, trunk, and perineal areas. Over time, the central parts of these lesions may necrotize and crust, eventually leading to the formation of ulcers, crusting, and hyperpigmentation. The progression of NME’s characteristic manifestations is often associated with the tumor’s excessive secretion of glucagon, which leads to a catabolic state causing deficiencies in essential fatty acids, amino acids, and minerals necessary for skin growth and turnover, ultimately resulting in epidermal protein deficiency and necrolytic disintegration (10). Literature suggests that deficiencies in amino acids or zinc may also trigger NME (9).

Compared to non-functional pancreatic neuroendocrine tumors, functional tumors generally have a better prognosis (11). This may be due to the hormone secretion by functional tumors leading to paraneoplastic syndromes, which can facilitate earlier diagnosis, providing clinicians with more direct indicators of tumor progression.

Despite this, diagnosing glucagonoma solely based on erythema can be challenging as NME is often misdiagnosed as other skin conditions, such as measles vasculitis, eosinophilic cellulitis with edematous erythema, or eczema with erosion (such as pemphigus). Skin biopsies and extracutaneous observations can assist in confirming the diagnosis. A careful review of 30 past cases of GCGN (**Table 1**) revealed that the initial symptom for the majority of patients was NME, typically starting in the lower limbs and gradually spreading to the entire body. Some patients also presented with anemia and blood glucose fluctuations, and there were cases of initial misdiagnosis due to a lack of specific clinical manifestations early on. Such patients were often discovered during routine health examinations or presented with abdominal discomfort, and were only diagnosed with GCGN after surgical removal (**Table 1**). Therefore, in addition to its specific clinical manifestations, imaging plays an indispensable role in diagnosing GCGN. Pancreatic CT is very useful for determining the precise location of the tumor, its relationship with surrounding organs, and metastatic status; glucagon level measurements are definitive for diagnosis (32); and the high expression of pancreatic somatostatin receptors is also crucial in diagnosis. Additionally, PET-CT scans hold certain advantages over CT and MRI in diagnostic contexts (33).

**Table 1:** A review of 20 cases of glucagon tumors.

Reference,Year	Age	Sex	First Symptoms	Initial Site	Anaemia	Blood Glucose Fluctuations	Tumor Location (Pancreatic)	Tumor Size (cm)	Stage	Liver Metastasis	Postoperative Recurrence
Bulent Dinc et al., 2009 (12)	53	F	NME	Lower limbs	Y	Y	Body	3.0	G2	Y	Postoperative death
Guido Poggi et al., 2009 (13)	52	M	NME	Lower limbs	N	N	Tail	2.9	G2	Y	None
Pablo Granero Castro et al., 2011 (14)	70	F	NME	Lower limbs	Y	Y	Tail	7.0	G2	N	None
Abolfazl Afsharfard et al., 2012 (15)	43	F	NME	Body	N	Y	Head	2.9	G2	N	Postoperative death
David Graham Watt et al., 2013 (16)	62	F	NME	Lower limbs	N	Y	Body	5.0	G2	N	Ovary
Sheng Fang et al., 2014 (17)	55	M	NME	Lower limbs	Y	Y	Tail	7.0	G2	N	None
Sheng-li Wu et al., 2014 (18)	44	F	NME	Head and face	Y	Y	Head	3.0	G2	N	None
JISHU WEI et al., 2015 (19)	50	F	NME	Body	Y	Y	Body	4.2	G2	N	None
Florentino de Araújo Cardoso Filho et al., 2015 (20)	56	M	NME	Lower limbs	Y	Y	Body	9.0	G2	N	None
Ashraf Al-Faouri et al., 2016 (21)	64	F	NME	Lower limbs	N	Y	Tail	5.0	G2	Y	None
Xu Han et al., 2016 (22)	60	M	NME	Lower limbs	N	Y	Tail	8.5	G2	N	None
Yun Gao et al., 2017 (23)	50	M	NME	Lower limbs	Y	Y	Head	3.0	G2	Y	None
Zhen-Xia Wang et al., 2019 (24)	48	F	NME	Lower limbs	Y	Y	Tail	5.8	G1	N	None
Mauricio Alvarez et al., 2020 (25)	44	F	NME	Lower limbs	Y	Y	Head	6.0	G2	Y	None
Mohamed K.M. Shakir et al., 2020 (26)	16	M	Asymptomatic	None	N	Y	Tail	2.6	G2	N	Bone
Shujuan He et al., 2021 (27)	57	F	NME	Head and face	Y	Y	Tail	2.0	G2	Y	Unsurgical
Wissal Abdelli et al., 2021 (28)	36	F	NME	Head and face	Y	Y	Tail	15.0	G2	N	None
Ouadi Yacine et al., 2022 (29)	44	F	NME	Lower limbs	N	Y	Body	4.9	G2	N	None
Sophia Garcia et al., 2023 (30)	47	F	NME	Lower limbs	Y	Y	Body	7.5	G2	Y	None
Shogo Amano et al., 2023 (31)	36	F	Asymptomatic	None	N	N	Body	1.5	G1	N	None

Compared to non-functional pancreatic neuroendocrine tumors (uFpNETs), functional pancreatic neuroendocrine tumors (FpNETs) generally have a better prognosis (11). This is likely because the hormones secreted by FpNETs cause paraneoplastic syndromes, providing clinicians with more direct indicators to assess tumor progression, thereby facilitating earlier diagnosis. It is noteworthy that surgical resection of the lesion often results in rapid symptom relief. Some patients experience tumor recurrence and metastasis post-surgery, leading to the re-emergence of the glucagonoma syndrome, which can be controlled again after subsequent debulking surgeries. Surgery, as a treatment option that can quickly alleviate symptoms, should be the first choice for managing functional neuroendocrine tumors when the patient’s general condition allows it. Therefore, radical surgical resection is the best approach for treating GCGN. However, most patients are diagnosed with metastases, making curative resection unfeasible.

For patients with pancreatic neuroendocrine tumor liver metastases who cannot undergo curative surgery due to diffuse hepatic spread, debulking surgery can be considered. Typically, debulking needs to achieve more than 90% tumor reduction to benefit the patient (34). Research by Morgan has shown that there is no significant difference in postoperative survival among patients with pancreatic neuroendocrine tumor liver metastases undergoing 100%, ≥90%, and ≥70% debulking, thus allowing the threshold for debulking to be relaxed to ≥70% (35). In the surgical treatment of the patient discussed here, considering the tumor’s invasion into the splenic artery and vein and liver metastasis, we not only resected the primary tumor in the pancreatic body and tail but also performed a splenectomy. While ensuring the patient’s postoperative residual liver tissue was sufficient for survival, we maximized the removal of the liver metastases. Comparing preoperative and postoperative imaging, over 90% of the liver metastases were successfully removed.

With the advent and application of new drugs and treatment modalities such as somatostatin analogs, anti-angiogenic agents, interventional embolization therapies (TAE), radiofrequency ablation, and peptide receptor radionuclide therapy (PRRT), outcomes similar to or better than surgical debulking can be achieved in terms of symptom control, tumor burden reduction, and improving long-term prognosis (36). The choice of medical treatments for pNETs should consider factors like the neuroendocrine functional status of the tumor, expression of somatostatin receptors, pathological grading, tumor staging, and the toxicity profile of the drugs. Currently used clinical somatostatin analogs (SSAs) include long-acting Octreotide (Octreotide LAR), Lanreotide, and Pasireotide, which are the first-line treatments for controlling hormone-related symptoms of functional neuroendocrine tumors. SSAs can also inhibit the growth of neuroendocrine tumors through mechanisms that include the activation of somatostatin receptors (SSTRs) inhibiting adenylate cyclase and ultimately suppressing the ERK signaling pathway. Activation of SSTRs also stimulates protein tyrosine phosphatase, which inhibits the mitogenic signaling pathway, and can suppress the PI3K-AKT pathway (37). A randomized, double-blind, prospective, placebo-controlled phase III clinical trial (PROMID study) showed that Octreotide LAR significantly extends the time to tumor progression (TTP). The median TTP in the Octreotide LAR treatment group was 14.3 months, compared to just 6.0 months in the placebo group (38).

Sunitinib is a domestically produced small molecule tyrosine kinase inhibitor (TKI) that targets the vascular endothelial growth factor receptors 1 and 2 (VEGFR1/2) and the colony stimulating factor-1 receptor (CSF-1R). In the SANET-p study, treatment with Sunitinib for advanced progressive pancreatic neuroendocrine tumors (PanNETs) resulted in a median progression-free survival (PFS) of 10.9 months (39).

Interventional embolization therapy is an important local treatment method for liver metastases in patients with pancreatic neuroendocrine tumors (pNETs), primarily including transarterial chemoembolization (TACE), transarterial embolization (TAE), and transarterial radioembolization (TARE). Most studies show no significant statistical differences between TACE and TAE in terms of tumor response, progression-free survival (PFS), and overall survival (OS), but TAE is better tolerated by patients (40, 41). Grozinsky et al. (42) studied 57 patients with NET liver metastases, where 53 showed significant symptom improvement, marked tumor response, and significant reduction in tumor marker levels after interventional treatments. The median survival time was 22 ± 18 months, with the most significant therapeutic effect observed in patients with pNET liver metastases.

In the early stages of the disease, the patient in this case exhibited only mild abdominal discomfort without significant skin lesions. Tumors in the pancreas and liver were discovered through enhanced abdominal CT scans. A liver tumor biopsy indicated a Grade 2 neuroendocrine tumor. Combining these findings with the patient’s imaging data, he was diagnosed with pancreatic neuroendocrine tumor metastases to the liver. Based on the patient’s overall condition, treatment began with transarterial embolization (TAE) combined with long-acting octreotide, with sunitinib added when liver metastases progression was noted. During conservative treatment, the patient gradually developed erythema, initially thought to be a drug rash from sunitinib. A biopsy of the erythema confirmed necrolytic migratory erythema. Considering the imaging results, liver metastasis biopsy pathology, elevated serum neuron-specific enolase, skin lesion pathology, and a serum glucagon level of 1138.3 pg/L—significantly above normal—it was conclusively diagnosed as GCGN with liver metastasis.

Initially, as the pancreatic tumor and liver metastases were detected early, hormone secretion from the tumors was controlled metabolically without causing paraneoplastic syndrome. As treatment progressed, the patient’s condition worsened with an increase in liver metastases and abdominal lymph node involvement. Subsequently, widespread necrolytic migratory erythema appeared without glucose fluctuations. Following surgical removal of the primary (pancreatic body and tail) and secondary tumors (liver metastases), symptoms improved significantly. Since the surgery did not achieve R0 resection, residual liver metastases remained, necessitating regular postoperative treatment with long-acting octreotide injections and oral sunitinib. As of the last follow-up (February 23, 2024), the patient’s general condition was satisfactory, although the liver tumors continued to progress without signs of paraneoplastic syndrome.

In summary, the initial symptom of GCGN is often skin lesions, and many clinicians rely on this clinical presentation for diagnosis. This reliance means the diagnosis of FpNETs often depends on significant hormone elevations and related clinical manifestations as the disease progresses, potentially overlooking early tumors with light loads and nonspecific or delayed clinical presentations, leading to misdiagnoses or missed diagnoses. After surgical removal of the primary tumor and most metastases, the reduction in tumor burden and hormone secretion leads to significant improvement in skin lesions. Our review of 30 GCGN cases found that nearly all patients whose initial symptom was NME saw rapid improvement in skin symptoms post-tumor resection. Reports indicate that in GCGN patients, tumor recurrence post-surgery led to the reappearance of NME-like lesions, which were alleviated again after a second resection.

This emphasizes the need for vigilance against potential tumor recurrence during treatment, especially in patients with multiple liver metastases who cannot undergo curative resection. Symptoms may reappear as the tumor progresses. Thus, regular follow-ups and clinic visits are necessary for such patients, with long-term treatment with SSA drugs for those at high risk of postoperative recurrence. Monitoring through imaging and serum glucagon levels is essential to dynamically assess the patient’s prognosis and adjust the treatment plan accordingly.

Through the analysis of this case and literature review, we suggest that early screening of serum hormones should be conducted for pNETs diagnosis to avoid misdiagnoses or missed diagnoses due to low early-stage hormone levels and mild or ambiguous clinical symptoms. For patients with higher hormone secretion levels and clearer clinical manifestations, surgery should be considered as soon as possible after ruling out contraindications.

## Conclusion

This report of a glucagonoma (GCGN) case enriches our understanding of the diagnostic and treatment modalities for this disease. In this case, the initial symptoms were atypical, and the tumor was discovered without the characteristic skin lesions and significant blood glucose fluctuations, which are uncommon in previous case reports, thus presenting challenges in diagnosis and treatment. The detailed record of this case will assist in managing similar future cases to improve patient prognosis. Additionally, this article reviews thirty related cases to explore the clinical characteristics associated with GCGN.

## Data Availability

The original contributions presented in the study are included in the article/supplementary material. Further inquiries can be directed to the corresponding author.
